# Precision medicine decision-making under pharmacogenomics in nursing: realizing value from limited evidence to scaled clinical pathways

**DOI:** 10.3389/fneur.2026.1808157

**Published:** 2026-06-29

**Authors:** Guoling Qin, Xing Luo, Xuanrun Hao, Haiting Xiao, Kunpeng Wang, Houjun Pang, Meilin Li, Chuan Pi, Sha Yang, Qixiong Zhang

**Affiliations:** 1 Department of Nursing, Dazhou Vocational College of Chinese Medicine, Dazhou, Sichuan, China; 2 Department of Pharmacy, Sichuan Provincial People's Hospital East Sichuan Hospital and Dazhou First People's Hospital, Dazhou, Sichuan, China; 3 Department of Pharmacy, Dazhou Maternal and Child Health Hospital, Dazhou, Sichuan, China; 4 Department of Clinical Laboratory Medicine, Southwest Hospital, Third Military Medical University, Shapingba, Chongqing, China; 5 Shigatse Branch, Xinqiao Hospital, Third Military Medical University, Shigatse, Xizang, China; 6 Department of Pharmacy, Personalized Drug Research and Therapy Key Laboratory of Sichuan Province, Sichuan Academy of Medical Science and Sichuan Provincial People's Hospital, School of Medicine, University of Electronic Science and Technology of China, Chengdu, China

**Keywords:** antidepressants, antipsychotics, individualized psychiatry, pharmacogenomics in nursing, precision medicine, psychiatric disorders

## Abstract

**Background:**

Inter-individual variability in efficacy and tolerability remains a major challenge in psychiatric prescribing. Pharmacogenomics (PGx) has been proposed as a practical tool to reduce avoidable drug-gene mismatches, particularly for antidepressants and selected antipsychotics metabolized by CYP2D6 and CYP2C19. However, the clinical value of PGx in psychiatry is uneven across drug classes, testing panels, and patient populations.

**Purpose:**

To promote the long-term integration of precision medicine and care, transforming digital PGx outcomes into tangible health benefits for patients.

**Methods:**

This article was a structured narrative review. PubMed, Embase, and CINAHL were searched for English-language literature published from January 2010 to January 2026, with emphasis on contemporary guidelines, pragmatic trials, randomized controlled studies, meta-analyses, and implementation studies relevant to psychiatric prescribing, nursing practice, and digital decision support. Search terms combined PGx-related keywords with psychiatry, antidepressants, antipsychotics, CYP2D6, CYP2C19, nursing, clinical pathway, electronic health record, and artificial intelligence concepts. We prioritized professional guidelines and primary comparative studies, and excluded preclinical studies, conference abstracts without sufficient methods, non-psychiatric applications, and commercial claims unsupported by peer-reviewed outcome data.

**Results:**

Current evidence supports PGx most clearly as an adjunctive prescribing tool for selected antidepressants and antipsychotics with established drug-gene guidance. Across depression trials, pooled estimates suggest modest improvements in remission or response, but the magnitude and durability of benefit vary substantially, and large pragmatic studies have shown smaller and non-persistent effects at endpoint. The patients most likely to benefit are those with prior treatment failure, troublesome adverse effects, concentration-sensitive medications, or complex polypharmacy with potential phenoconversion. By contrast, routine testing for all psychiatric patients is not supported by current evidence. Nurses can contribute meaningfully to medication history-taking, patient education, sample logistics, adherence support and structured monitoring, but implementation in routine care requires realistic attention to workforce capability, genetic literacy, report standardization, electronic health record integration and clinical decision support.

**Conclusion:**

PGx should be positioned as part of a broader precision-prescribing framework, which is beneficial in the field of psychiatry that is oriented toward the transformation of clinical practice.

## 1 Introduction

Psychiatric disorders are leading contributors to the global burden of disease (1). Lifetime prevalence estimates are approximately 20% for major depressive disorder (MDD) (2) and 0.7% for schizophrenia (3). Mental disorders affect nearly one in seven people worldwide (4). Although antidepressants and antipsychotics remain first-line interventions, substantial interindividual variability in treatment response and tolerability persists (5, 6). This variability contributes to prolonged illness (7), polypharmacy (8), poor adherence (9), and increased healthcare expenditures (10). Patients with severe mental illness also experience markedly reduced life expectancy (10–20 years), driven in part by medication-related adverse effects and undertreated medical comorbidities (11, 12). Yet the practical challenge in psychopharmacology is not only whether medication is indicated, but which drug to start, at what dose, and how to respond when benefit is delayed or adverse effects emerge.

Pharmacogenomics (PGx) has become one of the most visible strategies for reducing avoidable trial-and-error prescribing in psychiatry. Its appeal is straightforward: common psychotropics are metabolized through pathways in which genetic variation can alter drug exposure (13–15), and in some cases toxicity and patient clinical outcomes (16–18). With accumulating evidence, real-world and pragmatic studies suggest that pre-emptive PGx testing can reduce adverse drug reactions and related hospitalizations (19, 20).

To facilitate translation of PGx evidence into practice, multiple professional organizations and regulatory bodies have developed and continually updated implementation resources. Major sources include the Pharmacogenetics Knowledge Base (Pharm GKB; www.clinpgx.org), the Clinical Pharmacogenetics Implementation Consortium (CPIC; www.clinpgx.org), and other international guideline groups, as well as drug-label information from the U. S. Food and Drug Administration (FDA; www.fda.gov) and the European Medicines Agency (EMA; www.ema.europa.eu) (21–26). Guidelines are available for using PGx results when prescribing antidepressants (21–24), and PGx information is included in FDA labeling for many antidepressants (25). However, implementation remains challenging due to limited structured genotype fields in electronic health records (EHRs), inconsistent interpretation of reports (26), and heterogeneous clinician awareness and acceptance (27). Within this context, nurses are well-positioned to bridge implementation gaps and support value-based care. As frontline providers of medication management and patient monitoring, nurses routinely observe and document treatment response, adverse effects, and adherence behaviors. Nonetheless, the nursing literature has largely emphasized perceptions, education, and commentary (28, 29), with comparatively few empirical studies describing how nurses operationalize PGx in routine psychiatric practice.

**Figure 1:**
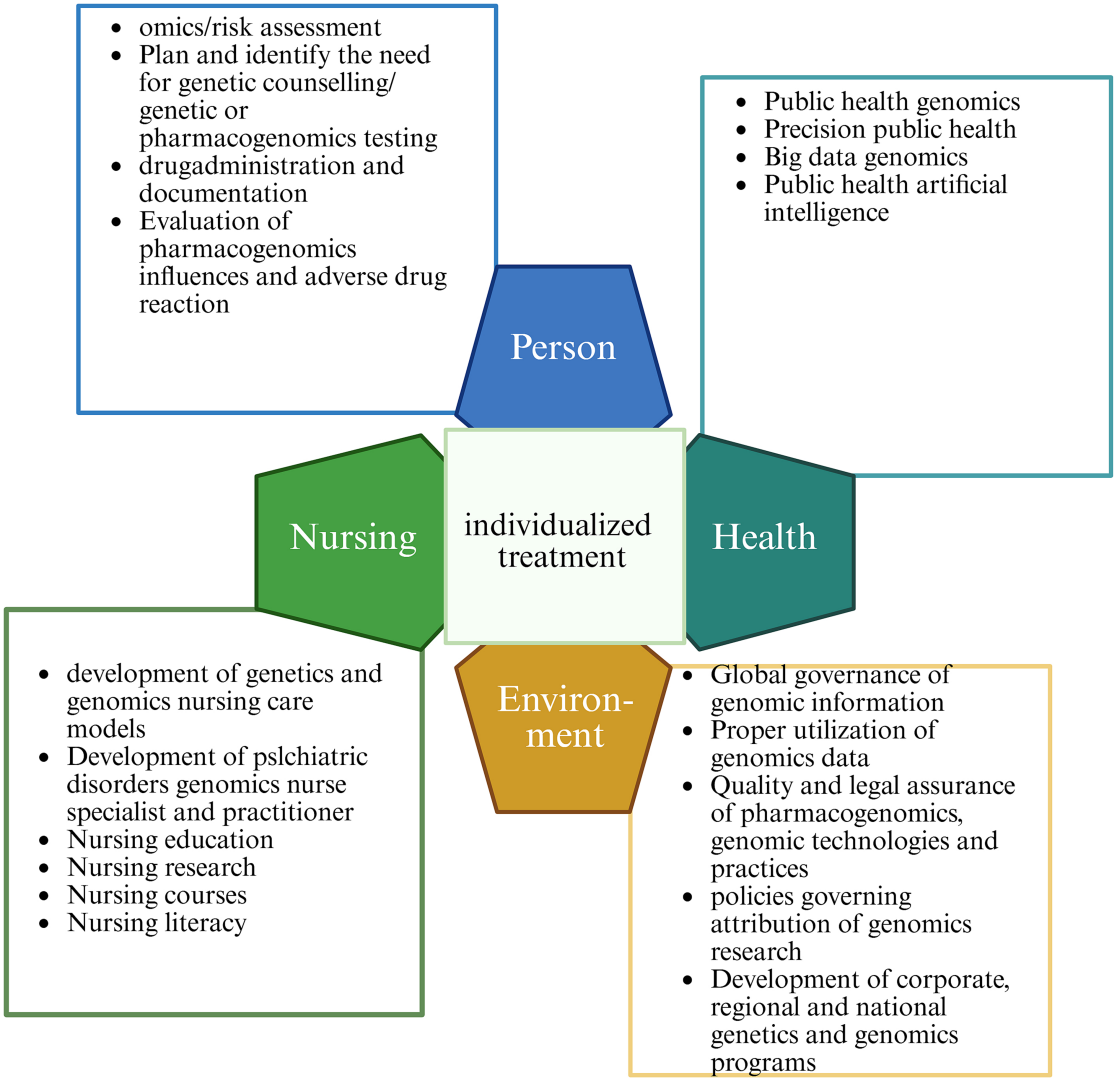
Emerging expanded psychiatric disorders nursing roles in PGx care. Adapted with permission from the reference (89). License: This is an open access article under the CC BY license (http://creativecommons.org/licenses/by/4.0/).

The purpose of this review is to reposition psychiatric PGx in a more transparent and clinically grounded framework. We examine the current evidence for antidepressants and antipsychotics, identify the patient groups most likely to benefit, summarize the most credible guideline-aligned gene-drug pairs, and discuss how PGx can be integrated into psychiatric care pathways. Particular attention is given to the nursing role, because patient education, adherence monitoring, adverse-effect surveillance, and continuity across care transitions are central to whether PGx information is actually used rather than merely generated. By revisiting the nursing meta-paradigm, psychiatric nurses can clarify the conceptual foundations of practice and delineate emerging roles in PGx-informed care (as shown in Figure 1).

## 2 Methods and scope

This article is a structured narrative review rather than a formal systematic review of effectiveness. To improve transparency, we prespecified three evidence domains: first, clinically actionable PGx guidance for psychiatric prescribing; second, comparative evidence on clinical utility, including randomized, pragmatic, and meta-analytic studies; and third, routine-care implementation issues, including nursing practice, clinical pathways, electronic health record integration, and decision support.

We searched PubMed, Embase, and CINAHL for English-language publications from 1 January 2010 to 31 January 2026. For implementation, nursing and digital health topics, particular emphasis was placed on literature from 2021 onwards to reflect current workflows and infrastructure. The core PubMed search concept was: (“pharmacogenom*” OR “pharmacogenetic*” OR “PGx”) AND (“psychiatr*” OR “depress*” OR “antidepress*” OR “antipsychot*” OR “schizophren*” OR “psychosis”) AND (“CYP2D6” OR “CYP2C19” OR “CYP2B6” OR “CYP3A4” OR “guideline” OR “trial” OR “meta-analysis”). For implementation-focused sections, the search was expanded with (“nurs*” OR “clinical pathway” OR “decision support” OR “electronic health record” OR “implementation” OR “artificial intelligence”).

We prioritized clinical guidelines, regulatory or professional recommendations, randomized controlled trials, pragmatic trials, systematic reviews, meta-analyses, and real-world implementation studies. Narrative reviews were used only to provide background context when primary evidence was limited. We excluded non-psychiatric PGx studies, preclinical reports, editorials, duplicate publications, conference abstracts that lacked sufficient methodological detail, and commercial materials without peer-reviewed clinical outcome data. Titles and abstracts were first screened for direct relevance to psychiatric prescribing or psychiatric service implementation, after which full texts were assessed for whether they contributed actionable information on prescribing, comparative benefit, or feasibility in routine care. When multiple reports described the same trial or panel, we preferentially cited the primary publication, prespecified secondary analyses, and the most recent guideline update.

## 3 Advancement in psychiatric PGx clinical evidence

### 3.1 The effectiveness of antidepressants and polymorphisms in metabolic enzyme genes

Antidepressant efficacy and adverse effects show substantial interindividual variability, much of which reflects pharmacokinetic differences driven by polymorphisms in drug-metabolizing enzymes. Selective serotonin reuptake inhibitors (SSRIs), serotonin–norepinephrine reuptake inhibitors (SNRIs), and tricyclic antidepressants (TCAs) are primarily metabolized by hepatic CYP450 enzymes, with CYP2D6 and CYP2C19 representing the most clinically actionable genes. As shown in Table 1, the evidence with the highest clinical applicability is primarily concentrated in the CYP2C19/CYP2D6-mediated pharmacokinetic pathways. In particular, CYP2C19 variation has been extensively studied for its impact on SSRIs such as citalopram and escitalopram. CYP2C19 ultra-rapid metabolizers (UMs) may exhibit increased clearance, potentially reducing exposure and therapeutic benefit. A large cohort study reported that among escitalopram-treated patients, both CYP2C19 UMs and poor metabolizers (PMs) were more likely to switch antidepressants early due to suboptimal efficacy or adverse reactions. Conversely, CYP2C19 PMs may develop elevated plasma concentrations on standard doses, increasing the risk of concentration-related toxicity, including sedation and QT prolongation (30). Accordingly, CPIC and DPWG guidelines recommend dose reduction or alternative agents for CYP2C19 PMs receiving citalopram/escitalopram. For sertraline, which is partially metabolized by CYP2C19, DPWG recommends limiting the maximum dose in CYP2C19 PMs.

**Table 1:** Summary of actionable psychiatric PGx gene-drug pairs.

Clinical domain	Key drug (s)	Gene	Evidence strength	Clinical dosage recommendation	References
Antidepressants	Imipramine, escitalopram, doxepin, clomipramine, citalopram, amitriptyline, trimipramine, sertraline	CYP2C19	High	CYP2C19 PM: reduce starting dose or use alternative SSRI (to avoid high levels). Monitor for sedation/QT prolongation.	(22)
	Clomipramine, desipramine, imipramine, fluvoxamine, nortriptyline, venlafaxine, trimipramine, paroxetine, amitriptyline, doxepin	CYP2D6	High	CYP2D6 PM: consider 50% dose reduction or switch to a non-CYP2D6 substrate antidepressant. (UM: may require higher dose.)	(22)
	Citalopram/es, sertraline, paroxetine, fluoxetine, fluvoxamine, venlafaxine	SLC6A4	No guideline (research only)	5-HTTLPR S allele: associated with lower SSRI response in some studies (esp. in Caucasians)-consider alternative strategy or monitor closely. Not clinically actionable by guidelines.	(22)
	Paroxetine	5HTR2A	No guideline (research only)	Common SNPs (e.g., 102 T/C) have been linked to SSRI efficacy in meta-analyses, but clinical use is not established.	(22)
	Multiple antidepressants (primarily focused on SSRIs/SNRIs/TCAs)	BDNF	No guideline (research only)	Val66Met (rs6265): associated with SSRI response in some studies, but not used in dosing guidelines.	(40)
	Multiple antidepressants (especially BBB-associated substrates)	ABCB1/ABCG2	No guideline (research only)	Efflux transporter variants may affect brain drug levels; currently not in prescribing guidelines.	(41)
Antipsychotics	Brexpiprazole, haloperidol, pimozide, aripiprazole, zuclopenthixol, risperidone	CYP2D6	High	CYP2D6 PM: substantially active moiety; reduce dose (~50% or more) for PMs. CYP2D6 UMs: may fail standard dosing; consider alternative. Pimozide (FDA): max 4 mg/d in CYP2D6 PMs	(42)
	Quetiapine, Olanzapine	CYP3A4	High	CYP3A4 PMs: consider lower quetiapine dose or alternate drug due to reduced clearance. Monitor for sedation.	(42)
	Second-generation antipsychotics like Clozapine, Risperidone, Olanzapine and Quetiapine	HTR2C/HTR2A	No guideline (research only)	HTR2C-759C/T variant: T allele linked to greater weight gain with some antipsychotics. No dosing change guideline; intensify metabolic monitoring if risk alleles.	(35, 52)
	Clozapine	HLA	No guideline (research only)	No genetic test required by current FDA rules (blood monitoring mandatory for all). Be vigilant for neutropenia, especially in known risk genotypes.	(53)

CYP2D6 variation has a more pronounced influence on TCAs and on certain SSRIs such as paroxetine. CYP2D6 UMs may clear the drug rapidly, risking reduced efficacy, whereas PMs exhibit impaired clearance and increased exposure. For example, amitriptyline concentrations in CYP2D6 PMs can exceed those in normal metabolizers (NMs) by more than two-fold, increasing anticholinergic adverse effects and cardiac toxicity (31). CPIC has provided genotype-based dosing recommendations for TCAs since 2016, including avoidance of standard dosing or substantial dose reduction in CYP2D6 PMs. Paroxetine is both highly dependent on CYP2D6 metabolism and a potent CYP2D6 inhibitor (32). Pharmacokinetic studies suggest that paroxetine exposure in CYP2D6 PMs may increase 3- to 5-fold, with higher discontinuation rates and intolerance; dose reduction or selection of an alternative antidepressant with minimal CYP2D6 dependence is therefore recommended (33). Importantly, not all putative genetic predictors of antidepressant response have reached an evidence threshold for routine clinical use.

Among non-CYP genes, SLC6A4 (encoding the serotonin transporter) and HTR2A (encoding a serotonin receptor) are among the most extensively investigated. The SLC6A4 promoter polymorphism 5-HTTLPR includes “short” and “long” alleles, with the long allele sometimes associated with enhanced SSRI response. Recent findings also indicate that demographic factors (such as ancestry and sex) may moderate observed associations (34). HTR2A includes common single nucleotide polymorphisms (SNPs), such as 102 T/C (rs6313) (35) and rs7997012G/*A. meta*-analyses have reported associations between these variants and antidepressant efficacy and tolerability (36, 37). Despite these signals, evidence remains inconsistent and insufficient to support actionable prescribing recommendations for SLC6A4 or HTR2A. Accordingly, the International Society for Psychiatric Genetics (2019) and CPIC have not recommended routine clinical use of these markers for antidepressant selection or dosing.

Additional candidate genes, including BDNF rs6265 (38) and transporters such as ABCB1 and ABCG2 (39), continue to be investigated. To date, evidence remains inadequate for clinical guideline integration (40, 41). Future work may improve predictive performance through polygenic models or machine-learning approaches that integrate multi-marker genetic data with clinical and environmental covariates.

### 3.2 The effectiveness of antipsychotics and polymorphisms in metabolic enzyme genes

Evidence for PGx-guided antipsychotic prescribing has expanded more recently than in antidepressant research. Many antipsychotics are metabolized by CYP2D6 and CYP3A4, and CYP2D6 polymorphisms are clinically relevant for agents such as risperidone, aripiprazole, and pimozide (42). Risperidone is converted by CYP2D6 to the active metabolite paliperidone (43), and CYP2D6 genotype influences parent-to-metabolite ratios and overall active moiety exposure. In a retrospective cohort study of Norwegian patients treated with risperidone (*n* = 725) or aripiprazole (*n* = 890) over nearly 14 years, CYP2D6 PMs and intermediate metabolizers (IMs) exhibited approximately 1.6- and 1.4-fold higher active moiety exposure compared with NMs, respectively (*p* < 0.0001). In routine care, dose reductions applied without genotype information were modest (19% for risperidone; 15% for aripiprazole), whereas genotype-based models suggested larger reductions (~40 and ~35%, respectively), indicating that experience-based adjustments may leave some patients overexposed (44). Longitudinal data further suggest that extreme CYP2D6 phenotypes may increase discontinuation risk. One study reported a 2.9-fold higher discontinuation rate among CYP2D6 UMs receiving risperidone relative to NMs, and a 1.9-fold increase among PMs. Similarly, CYP2D6 PMs or UMs were associated with increased discontinuation after 3 months of aripiprazole treatment, emphasizing the potential value of pre-emptive genotyping and accounting for strong CYP2D6 inhibitors that can induce phenoconversion (45). DPWG recommends reduced initial dosing and close adverse-effect monitoring when prescribing risperidone to CYP2D6 PMs; for CYP2D6 UMs, early switching to an antipsychotic not primarily metabolized by CYP2D6 may be considered if high-dose treatment is ineffective. The FDA also recommends that the maximum dosing of pimozide in CYP2D6 PMs not exceed half the standard maximum (46).

CYP3A4 contributes to the metabolism of antipsychotics such as quetiapine and lurasidone (42). While CYP3A4 genetic variability exists, functionally extreme phenotypes are relatively uncommon and variants such as CYP3A4*22 generally have modest effects (47). Thus, the CYP3A4 genotype is less frequently actionable than the CYP2D6, although concomitant use of CYP3A4 inhibitors remains clinically important.

Beyond pharmacokinetic genes, pharmacodynamic and immunogenetic factors have been explored. DRD2 is the principal target of most antipsychotics, and the ANKK1 Taq1A variant has been examined for associations with response and tardive dyskinesia risk (48). HTR2C polymorphisms have been linked to variability in negative symptom response and to adverse metabolic effects such as weight gain, with some sex-specific signals (49). However, evidence remains heterogeneous and insufficient for standardized dose adjustment protocols (50–52). Clozapine-associated agranulocytosis has been linked to HLA alleles, including HLA-DQB1 (126Q) and HLA-B (53), but routine genetic screening is not mandated, likely due to rarity and universal blood monitoring requirements. In mood stabilization, the association between HLA-B*15:02 and carbamazepine-induced Stevens-Johnson syndrome is well established in several Asian populations (54), and many jurisdictions recommend or require genotyping prior to initiation.

### 3.3 The clinical effect magnitude of PGx evidence and the potential beneficiary population

Evidence supporting PGx-guided treatment continues to grow. A meta-analysis reported higher remission rates with PGx-guided care relative to treatment as usual (TAU), with a pooled risk ratio (RR) of 1.54 (95% CI 1.07–2.21; *p* = 0.02) (55). A randomized controlled trial (*n* = 206) also found that guided care improved response (*p* = 0.032) and remission (*p* = 0.014) compared with TAU (56). Subsequent meta-analyses have generally replicated higher remission rates in MDD under PGx-guided prescribing (57–62). However, the PRIME Care trial, the largest PGx trial in depression, found small symptom-remission gains and non-persistent at the primary endpoint (63, 64).

Recent quantitative syntheses reinforce both sides of the picture. A 2024 meta-analysis of 15 randomized trials found that PGx-guided care was associated with an average 3.4% greater reduction in symptom severity, a 0.75-point greater reduction in Hamilton Depression scores, an 18% higher response rate and a 37% higher remission rate, with no significant reduction in discontinuation (60). However, most associations weakened or lost statistical significance when analyses were restricted to lower-bias studies, and the authors noted possible publication bias. The remission and response were more likely under PGx guidance, but emphasized wide variation between panels, studies and pooled estimates.

For most patients, any incremental benefit is likely to be modest, typically limited to low-single-digit differences in symptom improvement or small absolute gains in cumulative remission, rather than a wholesale change in the overall outcome trajectory. The patients most likely to derive clinically meaningful benefit are those with treatment-resistant or recurrent depression after prior inadequate response, those with previous dose-limiting adverse drug reactions, those with an existing or planned prescription involving a clear CYP2D6 or CYP2C19 mismatch, older or medically complex patients exposed to polypharmacy in whom drug–drug-gene interactions and phenoconversion may alter effective metabolizer status, and populations in which clinically relevant metabolizer genotypes are relatively common. By contrast, the added value of PGx is likely to be limited when poor outcomes are driven predominantly by non-adherence, insufficient follow-up, psychosocial burden, diagnostic uncertainty, or inadequate treatment intensity. Importantly, trial-level effect estimates for polypharmacy-defined subgroups remain limited or unspecified, and standardized analyses of time to response or time to remission are still relatively sparse. PGx is therefore best positioned, at present, as an adjunct decision-support tool for selected patients and selected prescriptions, rather than as a broadly transformative intervention for the majority of psychiatric patients.

## 4 Integrated application of PGx guidelines and clinical pathways

As evidence expands, embedding PGx into standardized clinical decision-making has become increasingly important. Clinical guidelines provide a cornerstone for implementation, and in recent years, multiple professional organizations and regulatory bodies have issued recommendations that collectively frame clinical use.

### 4.1 CPIC, DPWG, CPNDS, and RNPGx

CPIC and DPWG primarily provide pharmacotherapeutic recommendations that translate genotype or predicted phenotype into actionable prescribing guidance (65). CPIC standardizes genotype-to-phenotype terminology and publishes decision support tools designed for integration into clinical information systems. In psychiatry, CPIC has issued guidance for TCAs (21), carbamazepine/oxcarbazepine (66), and SSRIs (67). DPWG has evaluated gene-drug interactions since 2005 and provides graded management strategies (such as no adjustment, caution, dose adjustment, contraindication) based on the evidence (68, 69). For example, DPWG guidance recommends that CYP2C19 PMs should not exceed 37.5% of the normal maximum dose for sertraline, whereas no adjustment is required for CYP2C19 IMs or UMs (65). For antipsychotics, DPWG recommends alternative therapy or dose reduction for quetiapine in CYP3A4 PMs for certain indications, while no adjustments are suggested for other predicted phenotypes (42). Several European countries have integrated DPWG recommendations into national e-prescribing systems, enabling automated alerts and dosing guidance at the point of prescribing.

The Canadian PGx network for drug safety (CPNDS) and the French national PGx network (RNPGx) also provide recommendations regarding when genotyping should be performed in routine practice; RNPGx additionally specifies clinical contexts in which testing is recommended (65).

### 4.2 FDA, EMA, and AEMPS

Additional PGx information is provided through regulatory product documentation, including EMA-approved summaries of product characteristics (SmPCs) and FDA-approved drug labels (65). The proportion of drugs with PGx content in regulatory labeling has increased over time (70). Comparative analyses suggest moderate concordance in PGx recommendation levels between regulatory agencies; for example, the coincidence rate between the Spanish Agency for Medicines and Medical Devices (AEMPS) and the FDA has been reported as 45–65%, with ‘actionable’ guidance being most frequent (71). Direct-to-consumer (DTC) genetic testing has further amplified the visibility of PGx. In 2018, the FDA authorized 23andMe to market reports for selected pharmacogenetic variants, signaling regulatory recognition of DTC PGx information in consumer-facing contexts.

The current levels of evidence for PGx in psychiatry can be broadly categorized into three tiers: Tier 1 comprises pharmacokinetic biomarkers that have already been incorporated into prescribing recommendations; Tier 2 consists of safety risk biomarkers primarily used for enhanced monitoring; and Tier 3 includes exploratory pharmacodynamic biomarkers that are not yet sufficient to routinely guide prescribing. This article summarizes relevant gene-drug pairs based on the aforementioned PGx evidence and guidelines, as shown in Table 1.

Although prescribing decisions in psychiatry are typically physician-led, obtaining, interpreting, and operationalizing PGx results requires multidisciplinary collaboration involving laboratories, pharmacists, and nursing teams. Notably, most PGx guidance has been developed by physician- and pharmacy-led groups, with limited direct input from nursing organizations. PGx is the foundation of precision health, and nurse practitioners (NPs) must be prepared to integrate PGx science into clinical care (72).

## 5 Nursing-led PGx clinical pathway integration

Digital PGx implementation has been widely studied in oncology (73–75) and immune-related diseases (76), whereas empirical studies in mental health remain limited. Sulin Wu’s research indicates that high-quality cancer care is key to success (73). However, Kathleen A. Calzone’s online survey highlighted substantial variability in genomic literacy and infrastructure, with only one country reporting genetic/PGx competencies as requirements for general nurses (77). To better realize the value of precision medicine in mental illnesses, PGx research can be integrated into routine care processes.

### 5.1 Integration of PGx into nursing workflows

**Figure 2:**
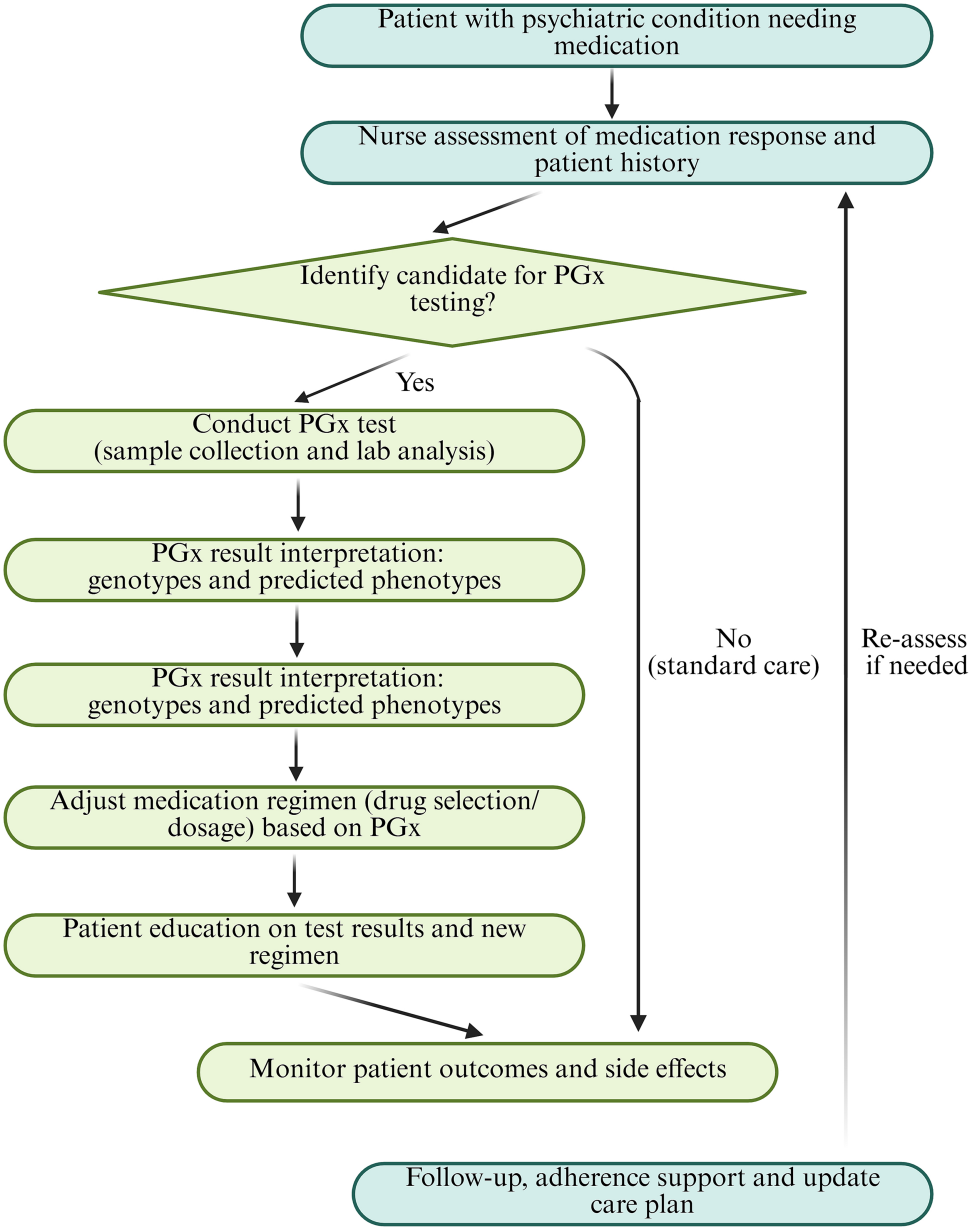
Scaled clinical pathways for integrating PGx into psychiatric medication management.

As shown in Figure 2, nurses have a pivotal role in psychiatric PGx clinical pathways. They are often the first members of the care team to collect medication and family histories, identify patients who may benefit from genetic testing, coordinate informed consent, and arrange sample collection. Once results become available, nurses may contribute to the preliminary interpretation of high-risk genotypes and participate in multidisciplinary case discussions to support the development of genotype-informed, individualized pharmacotherapy plans.

Nurses are also central to translating these recommendations into bedside care. This includes incorporating PGx-guided recommendations into nursing care plans, supporting regimen adjustments, establishing monitoring strategies, and explaining the clinical implications of test results and treatment decisions to patients and families. Such communication is essential for improving long-term medication adherence. During follow-up, nurses monitor treatment response and adverse effects, reinforce adherence, and update care records to ensure closed-loop use of genetic information and to reduce the risk of prescribing that is inconsistent with a patient’s genotype. These functions are particularly relevant in psychiatry, where therapeutic success depends not only on drug selection but also on continuity of care, adherence, and early recognition of medication intolerance.

Reasonable clinical pathway triggers include repeated non-response, troublesome adverse effects, prior prescribing conflicts with known gene-drug guidance, or polypharmacy likely to alter observed metabolism. Once testing is ordered, results should enter the electronic health record as discrete data rather than static PDF documents, be linked to point-of-care decision support, and be interpreted together with concomitant inhibitors, inducers, and clinical context. After treatment adjustment, follow-up should focus on symptom trajectory, tolerability, adherence and whether the treatment change produced a clinically meaningful benefit.

### 5.2 Implementation challenges

#### 5.2.1 Lack of information technology and decision support

Implementation of this workflow requires integration of PGx results into EHRs and the development of clinical decision support (CDS) tools. At present, many hospitals have not incorporated PGx results into EHRs systems (79), limiting the availability of automated alerts. Although CDS systems can provide individualized prescribing recommendations at the point of care, their development and maintenance are resource-intensive. In practice, the lack of mature CDS infrastructure remains a major technical barrier to the routine clinical use of PGx (78).

#### 5.2.2 Deficiencies in capability and talent systems

Nearly 98% of clinicians recognize that genetic variation can influence drug response, yet only approximately 10% report sufficient familiarity with PGx. PGx education among nurses is even more limited (80), and most regions worldwide have no formal requirements for PGx training in nursing curricula or professional development. Continuous education for both physicians and nurses is therefore needed to strengthen PGx literacy and interpretation across the multidisciplinary care team.

#### 5.2.3 Resource and workflow burden

The cost of PGx testing remains substantial, and reimbursement is often limited, which may discourage both patients and healthcare institutions from adopting the test. Incorporating genetic testing into routine care also introduces additional workflow steps, including counselling, sample handling, and result interpretation, which may further strain limited nursing resources. Studies have shown that many clinical teams regard testing costs and workflow complexity as major obstacles to PGx implementation. Policy incentives, institutional support, and phased implementation strategies (81) may help reduce these barriers.

#### 5.2.4 Ethical and compliance pressures

Ethical and privacy considerations are integral to PGx implementation. Nurses must ensure informed consent and protect data confidentiality, including by reaffirming patients’ willingness to participate during follow-up when appropriate. Safeguards are also needed to prevent misuse or disclosure of genetic information (81), which could have implications for insurance, employment, or social discrimination. Because relevant regulatory and legal frameworks are still evolving, these issues must be addressed explicitly when PGx is introduced into psychiatric practice.

Accordingly, minimum nursing competencies in psychiatric genomics should include the ability to recognize high-yield metabolic pathways for psychotropic medications; understand alerts for severe human leukocyte antigen-associated adverse drug reactions, particularly severe cutaneous reactions; communicate uncertainty without overstating the determinative value of genetic findings; document actionable phenotypes in the EHRs; and identify situations that require review by a pharmacist or prescribing clinician. These competencies are achievable, but only when supported by protected training time, standardized workflows, accessible CDS systems, and institutional leadership.

## 6 Future outlook

### 6.1 Artificial intelligence-assisted PGx interpretation and decision-making

There are already concrete examples of PGx-linked machine learning in psychiatry. Benrimoh et al. (82) trained a deep learning model using clinical trial data from 9,042 patients with depression to predict the probability of remission for 10 commonly used antidepressants; the model demonstrated good discriminatory power on the validation set (AUC ≈ 0.65) and improved the overall remission rate in simulated prescribing scenarios. Multi-omics models significantly outperform single-omics approaches in accurately predicting antidepressant response in patients with depression (83). However, there are few real-world AI studies involving nursing in psychiatry. Research in fields such as inflammatory bowel disease (84) and oncology (73) is limited, but it has been confirmed that high-quality cancer care is key to success. In the future, big data-driven “digital therapeutics” systems may emerge, inputting patient genomic and electronic health record data into AI algorithms to enable personalized drug recommendations—an approach that could also be effectively implemented in the nursing field.

### 6.2 DTC genetic testing and care management

The increasing availability of DTC genetic testing enables individuals to access PGx-related information without clinical mediation (85). While DTC testing may raise public awareness of PGx, it also increases the risk of misinterpretation and unsupervised medication changes. As frequent points of contact for patients, nurses can be prepared to counsel patients on appropriate interpretation, limitations, and safe use of genetic information. Nursing organizations can also contribute to policy discussions aimed at standardizing DTC testing practices and protecting consumer rights.

### 6.3 Digital health platform and care

Digital health tools—from interoperable EHR systems to mobile applications—can enable sustained PGx integration into nursing workflows (86). Effective implementation requires standardization, interoperability, and real-time updating of health data. Shared platforms can provide unified decision support accessible to clinicians, nurses, pharmacists, and other stakeholders. Mobile applications and telemedicine may further support PGx-guided self-management by allowing patients to upload PGx results, track symptoms, and receive algorithm-driven prompts; nurses can monitor trends via professional dashboards and initiate timely follow-up. Robust data security and privacy protections are essential, and evolving legal frameworks will continue to shape ethical standards for PGx-enabled nursing care.

### 6.4 Education, policy, and value identification

To meet the demands of precision medicine, nursing education must evolve to include genetics, pharmacology, data literacy, and medical ethics. Future NPs will need competencies in interpreting genomic data, participating in individualized treatment planning, and communicating effectively with patients and multidisciplinary teams. This transformation will require educational reform, continuing professional development, and certification pathways (87, 88). Payment and evaluation systems should also recognize nursing contributions to precision medicine by incorporating outcomes such as adherence support and early adverse-event detection into performance frameworks. Aligning incentives with nursing value will deepen engagement and accelerate adoption.

## 7 Conclusion

Integrating precision medicine with nursing is an inevitable trajectory in modern healthcare. Precision nursing should be viewed not as an adjunct but as a core component of precision medicine, enabling personalized, longitudinal, and context-sensitive care. Through advances in technology, education, data integration, and multidisciplinary collaboration, nursing can play a pivotal role in translating PGx science into improved patient outcomes. In mental health care, this integration may shorten trial-and-error periods, reduce adverse effects, and enhance recovery trajectories—realizing the central promise of precision medicine: transforming scientific progress into tangible improvements in health and well-being.
